# Glimpse into the Genomes of Rice Endophytic Bacteria: Diversity and Distribution of Firmicutes

**DOI:** 10.3389/fmicb.2016.02115

**Published:** 2017-01-05

**Authors:** Vasvi Chaudhry, Shikha Sharma, Kanika Bansal, Prabhu B. Patil

**Affiliations:** Bacterial Genomics and Evolution Laboratory, CSIR-Institute of Microbial TechnologyChandigarh, India

**Keywords:** plant-microbe interactions, endophyte, rice seeds, NGS, genomics, firmicutes, *Staphylococcus*, *Bacillus*

## Background

Endophytic bacteria inhabit within plant tissues without causing any evident damage to the host and play crucial roles in plant growth, development, fitness, and protection (Farrar et al., 2014; Truyens et al., 2015). These endophytic bacteria spend a portion of their life cycle inside plants and normally resides on intercellular spaces and gain carbohydrates, amino acids, and inorganic nutrients from plants (Bacon and Hinton, 2007). Despite their beneficial effects on plant growth and development, seed-borne endophytic bacteria are still largely unexplored. Recent developments in high-throughput technologies, such as next-generation sequencing (NGS), permit the investigation of endophytic microbiomes, facilitate sequencing of a larger number of bacteria and encourage *in depth* analyses of bacterial communities from taxonomy, phylogeny, and evolutionary studies (Kaul et al., 2016).

Genomes of endophytic bacteria encode all the information that is required for an organism to grow under a range of both favorable and unfavorable conditions depending on the plant habitats. Along with the house-keeping machinery, these bacterial genomes also encode genes that are required for their endophytic life style and plant beneficial properties (Hardoim et al., 2015; Sheibani-Tezerji et al., 2015). In the present study, whole genome sequencing of 21 rice seed endophytic bacterial species belonging to the phylum Firmicutes was performed to ascertain their phylogenetic position and to get clue of the genomic signatures for their adaptation to endophytic lifestyle. Genomic dataset of endophytic bacterial strains are valuable pool of information that provides insights into the diversity, distribution, and lifestyle associated genes of these endophytes associated with plants.

## Methods

### Isolation of bacteria from surface sterilized rice seeds

Rice seeds (Variety: Pusa Basmati 1121) were collected from rice field located in Kharar, Punjab (30.6755° N, 76.6723° E) for 3 years and processed in three independent batches for seed sterilization and isolation of endophytic bacteria. Endophytic bacteria were isolated from surface sterilized rice seeds by following the process: The hulls were removed from rice seeds using sterilized forceps, and the seeds (5 g) were put in sterile falcon tubes and washed with sterilized water for 1 min and then with 1% sodium hypochlorite solution for 5 min. The seeds were again washed with 75% ethanol for 1 min. After another wash with sterilized water five times, the surface sterilized rice seeds were crushed in sterile mortar and pestle and suspended in sterile saline solution (0.85% NaCl). The seeds suspension was incubated for 2 h at 28°C under shaking condition. After that, 100 μl of each of Direct, 10^−1^,10^−2^, 10^−3^, and 10^−4^ dilution in sterile saline was plated in duplicates onto Nutrient agar (NA); King's medium B (KMB); Glucose yeast chalk agar (GYCA); Tryptic soy agar (TSA); Peptone sucrose agar (PSA) supplemented with 0.01% cycloheximide. The confirmation of surface sterilization was conducted by spreading the last water wash as well as placing the washed rice seeds onto different media plates. Further, the isolates were identified by 16S rRNA gene PCR using 16S rRNA universal primers (27F and 1492R) and 16S rRNA gene sequence was used for characterization using EzTaxon server (http://www.ezbiocloud.net/eztaxon; Kim et al., 2012) prior to whole genome sequencing. For preservation, 15% glycerol stocks of the pure culture of each isolate was prepared and maintained at −80°C.

### Whole genome sequencing (WGS) and data collection

Endophytic bacteria belong to Firmicutes phylum were revived from −80°C stocks onto NA plates and genomic DNA was isolated using Zymo ZR- Fungal/bacterial DNA isolation kit as per the instruction manual. The quality of the genomic DNA was assessed using agarose gel electrophoresis. DNA concentrations were estimated using a Nanodrop spectrophotometer ND-100 (Thermo Fisher Scientific, USA) and Qubit 2.0 fluorometer (Invitrogen, USA), with a double-stranded DNA High Sensitivity (dsDNA HS) Assay Kit. Both the ratio A260/280 and gel electrophoresis were used to ascertain the quality and purity of DNA samples. The input of 1 μg of genomic DNA from each sample was taken and standard protocol for the Nextera XT DNA (Illumina, San Diego, CA) sample preparation kit was used for library construction. The purified fragmented DNA was used as a template for a limited cycle PCR using Nextera primers and index adaptors. Cluster generation and sequencing of libraries were performed on the Illumina MiSeq platform (Illumina, San Diego, CA) with a 2 × 250 paired-end run.

### Genome assembly and annotation

Demultiplexing, FASTQ generation and adapter trimming in raw sequence reads were automatically performed by Illumina- MiSeq software. The paired-end raw reads containing FASTQ files were assembled into contigs. Total number of library reads, number of contigs, genome size, G+C content, and total coverage were analyzed using CLC Genomics Workbench software version 7.0.3. Complete 16S rRNA sequences were extracted from the assembled genomes using RNAmmer 1.2 server (Lagesen et al., 2007) and characterized by EzTaxon server. tRNA was calculated by tRNAScan-SE. Sequences were annotated using NCBI Prokaryotic Genome Annotation Pipeline (http://www.ncbi.nlm.nih.gov/genome/annotation_prok/) and Rapid Annotation System Technology (RAST) pipeline (Aziz et al., 2008).

### Genome based taxonomy and phylogenomics

Taxonomic relationship among the endophytic bacterial isolates was deduced using 16S rRNA gene which is considered as the important tool for taxonomic and phylogenetic analysis (Mizrahi-Man et al., 2013). In the era of genome based taxonomy, the 16S rRNA gene is still used for preliminary bacterial typing because it is present in at least one copy in every bacterial genome and its conserved regions enable simple sample identification (Land et al., 2015). Therefore, firstly, the 16S rRNA sequences were used for characterization using EzTaxon server. Further, for genome similarity assessment, BLAST-based average nucleotide identity (ANIb) and Genome to Genome Distance calculator or digital DNA-DNA hybridization (dDDH) values were used. Pairwise ANI was calculated using JSpecies (Richter and Rosselló-Móra, 2009) and dDDH (Auch et al., 2010) was calculated using web tool GGDC 2.0.

### Data deposition

The assembled genome sequences (in FASTA format) of all 21 Rice seed bacterial endophytes is deposited in NCBI GenBank. Assembly statistics and accession numbers of the sequenced genomes are mentioned in Table 1. Figshare link to download genomes in FASTA format for single point access: https://figshare.com/s/6deae778bbdb90c6563c.

**Table 1:** **Genome assembly statistics and annotation features of bacterial endophytes of rice and their accession numbers**.

**S. no**.	**Strain name**	**Organism**	**Size (Mb)**	**No. of contigs**	**N50 (bp)**	**Coverage**	**GC (%)**	**CDS**	**tRNA**	**rRNA**	**NCBI GenBank accession no**.
1	RE1.1	*Bacillus subtilis* RE1.1	4.16	291	69,344	30x	43.3	4369	50	3	LWJX00000000
2	RE1.3	*Bacillus subtilis* RE1.3	4.18	194	125,761	39x	43.2	4362	68	3	LWJY00000000
3	RE1.4	*Bacillus subtilis* RE1.4	4.12	90	149,496	42x	43.4	4248	73	8	LWJZ00000000
4	RE1.58	*Bacillus licheniformis* RE1.58	4.44	233	60,032	37x	45.5	4598	72	5	LWKC00000000
5	RE1.59	*Bacillus licheniformis* RE1.59	4.45	143	88,085	41x	45.6	4567	68	3	LWKD00000000
6	RE1.60	*Bacillus licheniformis* RE1.60	4.43	279	54,920	39x	45.5	4614	67	5	LWKE00000000
7	RE1.61	*Bacillus licheniformis* RE1.61	4.44	209	76,140	40x	45.6	4591	77	4	LWKF00000000
8	RE1.51	*Staphylococcus warneri* RE1.51	2.57	115	54,604	69x	32.5	2447	49	4	LWJM00000000
9	RE1.52	*Staphylococcus warneri* RE1.52	2.57	101	62,881	76x	32.5	2451	52	3	LWJN00000000
10	RE2.7	*Staphylococcus hominis* RE2.7	2.2	102	39,227	74x	31.4	2100	59	4	LWJO00000000
11	RE2.8	*Staphylococcus hominis* RE2.8	2.2	145	38,112	69x	31.3	2113	58	3	LWJP00000000
12	RE2.10	*Staphylococcus hominis* RE2.10	2.21	161	30,950	66x	31.3	2134	60	3	LWJR00000000
13	RE2.24	*Staphylococcus equorum* RE2.24	2.87	142	44,640	46x	32.9	2780	47	3	LWJS00000000
14	RE2.35	*Staphylococcus equorum* RE2.35	2.68	98	65,908	65x	33	2542	54	3	LWJU00000000
15	RE2.40	*Staphylococcus equorum* RE2.40	2.78	91	65,036	74x	32.9	2649	46	5	LWJW00000000
16	SE3.10	*Staphylococcus cohnii* SE3.10	2.58	106	67,071	64x	32.3	2351	53	6	JRVU00000000
17	SE4.1	*Staphylococcus cohnii* SE4.1	2.58	58	101,685	72x	32.3	2346	56	6	JRVV00000000
18	SE4.2	*Staphylococcus cohnii* SE4.2	2.57	89	97,298	65x	32.3	2353	54	5	JRVW00000000
19	SE4.3	*Staphylococcus cohnii* SE4.3	2.57	124	49,541	67x	32.3	2343	57	6	JRVX00000000
20	SE4.4	*Staphylococcus cohnii* SE4.4	2.57	134	46,787	56x	32.3	2333	55	5	JRVY00000000
21	SE4.5	*Staphylococcus cohnii* SE4.5	2.58	98	52,365	47x	32.3	2346	55	5	JRVZ00000000

### *In silico* mining of important genes

RAST annotated genomes was used to search important genes responsible for plant adaptation and plant protection.

## Data interpretation

### Isolation and identification

A total of 21 bacterial endophytes isolated from rice seeds (Variety: Pusa Basmati 1121). The isolates were identified based on 16SrRNA gene for initial characterization prior to whole genome sequencing and their identification using EzTaxon server is summarized in Table S1.

### Genome sequencing and phylogenomic inference

**Figure 1:**
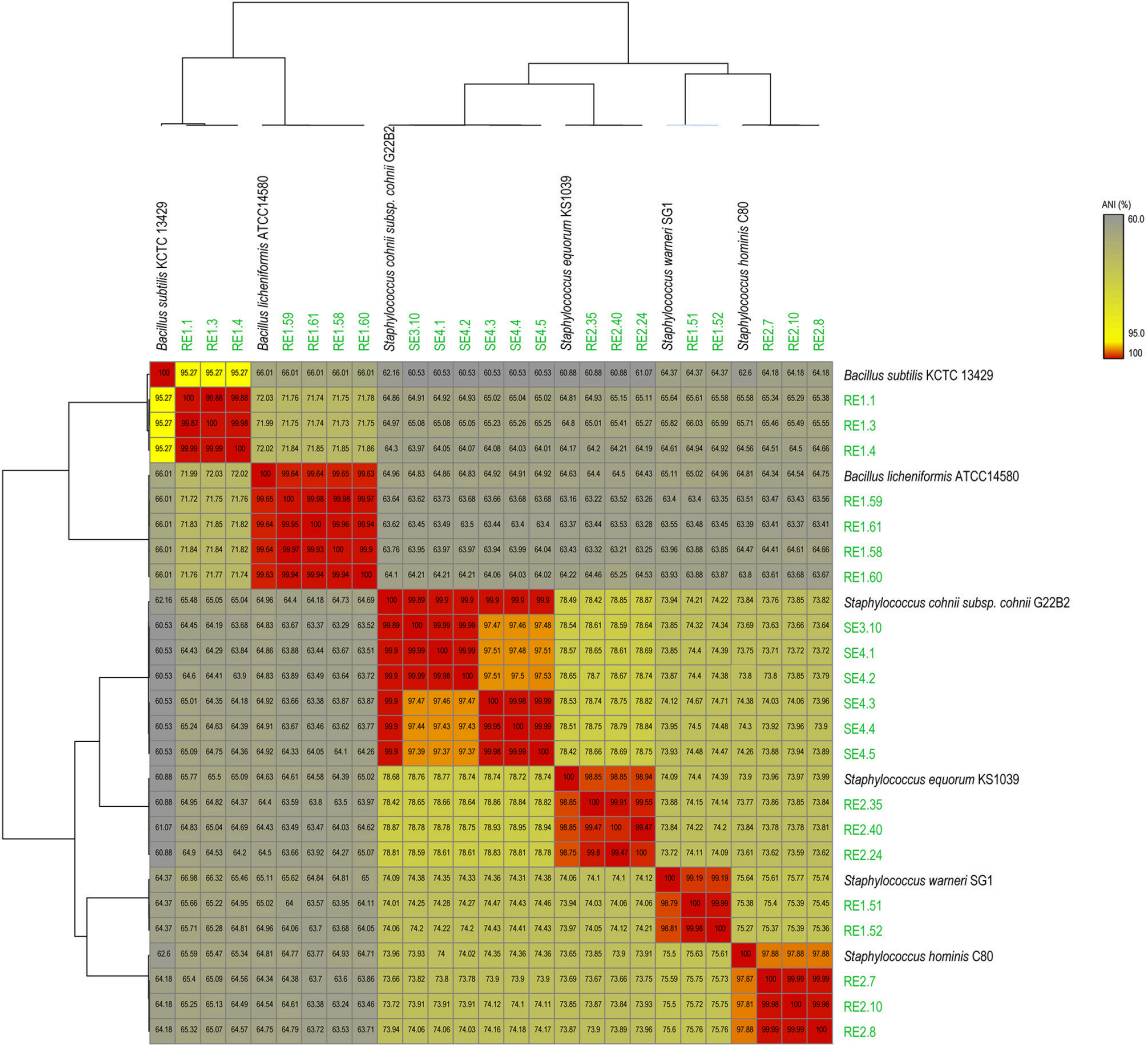
**Average Nucleotide Identity (ANI) heat-map showing the 21 rice bacterial endophytes in our dataset, along with genomes of best matches from NCBI genome database, indicating distribution of 6 species based on >95% ANI values cutoff**.

Sequence data generated for each strain (Table 1) was *de novo* assembled with coverage ranging from 30x to 76x. Analysis based on complete 16SrRNA gene sequence extracted from the whole genome sequences assigned the bacterial isolates into 2 distinct genera and 6 species (Table S1). To gain better taxonomic resolution, all the 21 bacterial isolates have been validated by species delineation cut-off of >95% ANI values and >70% dDDH values (Richter and Rosselló-Móra, 2009; Auch et al., 2010). Genome sequence of reference strains were taken from NCBI database. The ANI value heatmap and dDDH values of 21 endophytes genomes with Type strain/reference strain genome are depicted in Figure 1 and Table S2, respectively. Description of the strains assigned to 2 different genera is provided as follows:

### Genus: *Staphylococcus*

*Staphylococcus* is cocci shaped gram-positive member of phylum Firmicutes and 14 different strains were selected for sequencing from this genus belonging to 3 different year rice seeds sample lots. 16S rRNA sequences have assigned them to four different species with more than 99% identity to *Staphylococcus warneri* (RE1.51 and RE1.52), *Staphylococcus hominis* (RE2.7, RE2.8, and RE2.10), *Staphylococcus equorum* (RE2.24, RE2.35, and RE2.40), and *Staphylococcus cohnii* (SE3.10, SE4.1, SE4.2, SE4.3, SE4.4, and SE4.5). Moreover, ANI values also supported the species distinction between the four groups as they are above the cut-off (>95%) for species delineation (Figure 1). Further dDDH values of all the strains were more than 70% which further confirms their species identity (Table S2). Both ANI and dDDH resolved *Staphylococcus* species up to subspecies/strain level which were not possible with 16S rRNA gene sequences based analysis. This suggests that atleast 4 species of *Staphylococcus* inhabits rice seeds.

### Genus: *Bacillus*

*Bacillus* is a genus of gram-positive, rod-shaped bacteria and a member of the phylum Firmicutes. Seven isolates from different years of rice seed lots belong to two different species, *Bacillus subtilis* (RE1.1, RE1.3, and RE1.4), and *Bacillus licheniformis* (RE1.58, RE1.59, RE1.60, and RE1.61) with more than 99% identity of 16SrRNA gene sequence identity. ANI and dDDH values of RE1.58, RE1.59, RE1.60, and RE1.61 of >99 and >97% with type strain genomes unequivocally establish their species identity as *B. licheniformis* (Figure 1; Table S2). However, in case of RE1.1, RE1.3, and RE1.4, ANI value with type strain of *B. subtilis* was 95.2% that is close to borderline of species demarcation, and dDDH values of 48.9% also showed that it is closely related to *B. subtilis* and may belongs to separate species.

### Genome mining of plant beneficial traits

From RAST analysis, it was observed that more than 100 genes in the genomes of *B. subtilis* and *B. licheniformis* strains whereas more than 70 genes were present in genomes of *S. warneri, S. hominis, S. equorum, and S. cohnii* were identified that are responsible for stress tolerance. Majority of these genes were responsible for protection against oxidative stress and rest were encoded to cope with heat and osmotic stresses. Moreover, all the endophytes genomes of *Staphylococcus* and *Bacillus* species contain genes responsible for auxin biosynthesis and siderophores production. Full details of these genes are available in Table S3. It was reported that members of genus *Bacillus* are most commonly found as endophytic bacteria in plants. Moreover, they play important role as a biocontrol agent against phytopathogens, stimulates plant growth, and also produce plant growth hormones such as auxin and gibberellin, as well as able to ameliorate drought stress (Forchetti et al., 2007). There are also several reports in literature where members of *Staphylococcus* were documented as endophytes such as in rice seed (Chaudhry and Patil, 2016), maize kernels (Liu et al., 2012), grapevine, and hybrid spruce (Collins et al., 2004).

In the present study, our goal was to generate genomic data resource of endophytic bacteria associated with healthy rice seeds (var. Pusa Basmati 1121) from India. As representatives, dominant members belonging to Firmicutes phylum were selected. Our genomic dataset which comprised of *Staphylococcus* and *Bacillus* genera and their phylogenomic analysis reported here will serve to catalyze future studies by providing a new lens to study their endophytic lifestyle and will help in deciphering novel biological insights of endophytic bacteria-host plant relationships.

## Author contributions

VC carried out isolation, characterization, and preservation of bacterial endophytes; VC, SS, and KB performed QC and DNA library preparation. VC and SS performed genome sequencing, analysis, and submission; PP and VC designed of the study and interpretation of data. PP coordinated the study and applied for funding. VC wrote the manuscript. All authors reviewed and approved the manuscript.

### Conflict of interest statement

The authors declare that the research was conducted in the absence of any commercial or financial relationships that could be construed as a potential conflict of interest.
